# *Pseudostellaria heterophylla* Polysaccharide Increases Colonic Mucus Thickness and Bacterial–Epithelial Distance in HFD/STZ-Induced Diabetic Mice

**DOI:** 10.3390/cimb48101093

**Published:** 2026-10-21

**Authors:** Yongjun Kan, Yingying Liu, Li Zhao, Chang Jiang, Wenxiong Lin, Wensheng Pang, Juan Hu

**Affiliations:** 1 Institute of Materia Medica, Fujian Academy of Chinese Medical Sciences, Fuzhou 350003, China; kyj@fjtcm.edu.cn (Y.K.); 1181132006@fjtcm.edu.cn (L.Z.); fjjc123@hotmail.com (C.J.); 2 Fujian Key Laboratory of Agroecological Processing and Safety Monitoring, Fujian Agriculture and Forestry University, Fuzhou 350002, China; lyy@fjtcm.edu.cn (Y.L.); lwx@fafu.edu.cn (W.L.); 3 School of Pharmacy, Fujian University of Traditional Chinese Medicine, Fuzhou 350122, China; pws@fjtcm.edu.cn

**Keywords:** *Pseudostellaria heterophylla*, polysaccharide, HFD/STZ-induced diabetes, colonic mucus, bacterial spatial localization

## Abstract

*Pseudostellaria heterophylla* is a medicinal plant whose polysaccharides have shown metabolic and intestinal effects in animal studies. Mucus injury and bacterial encroachment are associated with metabolic disease. We evaluated colonic mucus morphology and bacterial spatial localization after PF40 treatment in male mice subjected to a high-fat diet and streptozotocin (HFD/STZ). Mice received PF40 (400 mg/kg/day) or vehicle for 6 weeks under shared dietary conditions. In exploratory comparisons, PF40-treated mice exhibited a thicker retained colonic mucus band, greater mucin 2-positive area and greater bacterial–epithelial distance. Colonic *Agr2* expression and occludin-positive area were higher, whereas circulating barrier-related markers and colonic inflammatory mediators were lower. Fasting glucose and homeostasis model assessment of insulin resistance were also lower. Fasting insulin was lower by Welch testing, whereas oral glucose tolerance test area under the curve did not differ significantly in the primary exact rank test; significance for both depended on the method. A separate fluorescein isothiocyanate–dextran pilot (*n* = 3 per treatment group) was inconclusive and requires validation in a larger cohort. These findings identify colonic mucus morphology and bacterial spatial localization as treatment-responsive phenotypes in this model. These concurrent observations do not establish whether mucus and spatial changes mediate barrier, inflammatory or glucose outcomes.

## 1. Introduction

Type 2 diabetes mellitus (T2DM) is a chronic metabolic disorder characterized by insulin resistance, progressive pancreatic β-cell dysfunction, and persistent hyperglycemia. Its microvascular and macrovascular complications impose a substantial disease burden [1]. Beyond classical metabolic organs, including the pancreatic islets, liver, skeletal muscle, and adipose tissue, the intestinal barrier connects the luminal environment to systemic metabolic homeostasis [2]. Hyperglycemia can reprogram intestinal epithelial transcription, disrupt cell junctions, and increase the risk that microbial products enter the circulation [3]. Intestinal barrier injury may therefore result from hyperglycemia and metabolic dysfunction. It may also increase the entry of microbial molecules into the mucosa and circulation, thereby promoting chronic low-grade inflammation and aggravating insulin resistance. In particular, entry of gut-derived lipopolysaccharide (LPS) into the circulation produces metabolic endotoxemia and can sustain innate immune and inflammatory signaling. This process is considered an important link between intestinal barrier dysfunction and abnormal glucose and lipid metabolism [4,5].

The intestinal barrier comprises interdependent components, including the mucus layer, epithelial cell junctions, antimicrobial molecules, and mucosal immunity. The colonic mucus layer lies between the luminal microbiota and epithelial cells, forming the first physical barrier that limits direct host–microbial contact. The gel-forming mucin 2 (MUC2), secreted by goblet cells, is the principal structural component of colonic mucus. The dense inner MUC2 layer restricts bacterial access to the epithelium, whereas the looser outer layer provides a habitat for some commensal microorganisms [6,7,8]. Mucus barrier structure and function depend on dynamic processes that include mucin synthesis, secretion, expansion, renewal, degradation, and glycosylation [9,10,11,12]. Bacterial–epithelial distance complements mucus thickness and MUC2-positive area by describing microbial proximity to the epithelial surface.

Mucus barrier injury and bacterial encroachment toward the epithelium are closely associated with metabolic disease. Dietary emulsifiers disrupt the mucus–bacteria interface in mice, promote bacterial encroachment, and induce low-grade intestinal inflammation and metabolic syndrome [13,14]. Dietary fiber deprivation can instead drive gut microorganisms to consume host mucin glycans, thereby eroding the colonic mucus barrier [15]. In humans, colonic bacterial–epithelial distance is inversely correlated with fasting blood glucose and glycated hemoglobin [16]. Obesity-associated microbiota can also reduce the growth rate and increase the penetrability of colonic mucus [17,18]. More recent multi-omics evidence links microbial carbohydrate metabolism to host insulin resistance and inflammatory phenotypes [19]. Together, these observations implicate the mucus barrier and microbial spatial organization in metabolic dysfunction.

Natural polysaccharides can reach the distal intestine and may affect the colonic mucus barrier through biophysical interactions, microbial fermentation, and host epithelial and immune responses [20]. Polysaccharides are important bioactive constituents of *Pseudostellaria heterophylla* (Miq.) Pax. PF40 is a *P. heterophylla* polysaccharide fraction with a molecular weight range of 50–210 kDa. Previous studies reported glucose-lowering, insulin-related and inflammatory effects in diabetic animals [21], and small-intestinal mucosal and immune findings [22]. These studies established a metabolic and general mucosal context for PF40. Colonic mucus thickness, MUC2-positive area and bacterial–epithelial distance characterize complementary aspects of mucus morphology and microbial proximity to the epithelium.

We therefore examined colonic mucus morphology and bacterial–epithelial spatial localization together to determine whether PF40 treatment was associated with changes in both phenotypes under the same HFD/STZ conditions. We hypothesized that PF40-treated mice would show a thicker retained colonic mucus band, larger MUC2-positive area and greater bacterial–epithelial distance than vehicle-treated mice. Both treatment groups shared the XTHF60/STZ background. We assessed these spatial and histological endpoints alongside mucus-related gene expression, fasting metabolic measures and oral glucose tolerance test (OGTT) area under the curve (AUC). Additional measures included areas positive for zonula occludens-1 (ZO-1) or occludin, circulating barrier-related markers, colonic inflammatory mediators and a separate fluorescein isothiocyanate–dextran (FITC–dextran) pilot.

## 2. Materials and Methods

### 2.1. Animals and Housing Conditions

Specific pathogen-free male C57BL/6J mice (6 weeks old; 18–22 g) were purchased from Xiamen Fudexin Biotechnology Co., Ltd. (production license: SCXK (Fujian) 2025-0001; Xiamen, China). Animals were housed in a specific pathogen-free barrier facility at 22 ± 2 °C and 50–60% relative humidity under a 12 h light/dark cycle, with ad libitum access to food and water. Experiments began after a 1-week acclimation period. All animal procedures were approved by the Laboratory Animal Ethics Committee of the Fujian Academy of Chinese Medical Sciences (approval No. FJACMS-SL-20260027) and followed relevant standards for laboratory animal care and use, including the Animal Research: Reporting of In Vivo Experiments (ARRIVE) 2.0 reporting principles.

Male mice were selected for this initial study because HFD/low-dose STZ metabolic responses differ by sex. Racine et al. observed fasting hyperglycemia, hyperinsulinemia and insulin resistance in male but not female C57BL/6J mice [23]. Casimiro et al. provide supporting evidence for sex-dependent HFD responses [24]. Racine et al. used STZ at 30 mg/kg/day for 3 days, whereas we used a single 35 mg/kg injection. Female responses to the present regimen require direct evaluation.

### 2.2. HFD/STZ Model Induction, Randomization and Experimental Cohorts

After acclimation, mice were randomly assigned to a non-diet-matched healthy reference group (CON, *n* = 10) or a model-induction cohort (*n* = 25). The CON group received an irradiated maintenance compound feed for adult mice and rats (Shanghai SLAC Laboratory Animal Co., Ltd., Shanghai, China). The manufacturer lists wheat and corn as energy sources; fish meal, chicken meal, and expanded soybean meal as protein sources; soybean oil as the fat source; bran and alfalfa as fiber sources; and added amino acids, vitamins, and minerals. The model-induction cohort received the purified XTHF60 high-fat diet (Jiangsu Xietong Pharmaceutical Bio-engineering Co., Ltd., Nanjing, China) for 4 weeks. XTHF60 provided 60.2% of energy from fat, 20.1% from protein, and 19.7% from carbohydrate; most fat was derived from lard. After high-fat feeding, mice in the model-induction cohort received an intraperitoneal injection of streptozotocin (STZ; 35 mg/kg body weight; Cat. No. S0130, Merck, Darmstadt, Germany) freshly prepared in 0.1 mol/L citrate–sodium citrate buffer (pH 4.5). The CON group received an equal volume of buffer.

The manufacturer-listed XTHF60 formulation comprised casein (200.00 g), L-cystine (3.00 g), corn starch (0 g), maltodextrin (125.00 g), sucrose (72.80 g), cellulose (50.00 g), soybean oil (25.00 g), lard (245.00 g), mineral mixture S10026B (50.00 g), vitamin mixture V10001C (1.00 g), choline bitartrate (2.00 g), and Food, Drug, and Cosmetic (FD&C) Blue No. 1 (0.05 g), for a listed total of 773.85 g and 4037.20 kcal (5.2 kcal/g).

On days 7 and 14 after STZ injection, mice were fasted for 12 h with free access to water. Blood was collected from the tail tip the following morning, and fasting blood glucose (FBG) was measured using an EZ3 glucometer and compatible test strips (Acon Biotech (Hangzhou) Co., Ltd., Hangzhou, China). Model establishment required FBG ≥ 11.1 mmol/L at both measurements. Nineteen mice met this criterion. These mice were stratified by FBG and body weight and then randomly allocated within strata to the model group (MOD, *n* = 9) or PF40 treatment group (PF40, *n* = 10). Post-randomization, pretreatment FBG and body weight were recorded at study week 6, before the first dose (Table S5). Body weight was followed weekly during treatment (study weeks 7–12; Table S6).

Investigators performing Alcian blue/periodic acid–Schiff (AB/PAS), immunofluorescence, and fluorescence in situ hybridization (FISH) image analyses were blinded to group allocation. Other experimental procedures and statistical analyses were not blinded.

The commercial maintenance feed and the defined-ingredient XTHF60 formulation differed in their ingredient matrix, not only in fat content. CON was therefore a non-diet-matched reference, rather than a control for the separate effects of dietary fat, STZ exposure or diabetes. The inferential treatment comparison was restricted to MOD versus PF40, which underwent the same model-induction procedure and remained on XTHF60 throughout treatment. CON outcomes are reported separately in Supplementary Figures and descriptive tables without inferential comparisons against MOD or PF40.

#### 2.2.1. Main Analysis Cohort

Before endpoint testing, animals were divided into a main analysis cohort (CON-main, *n* = 7; MOD-main, *n* = 6; PF40-main, *n* = 7) and a separate FITC–dextran pilot cohort. Main-cohort animals contributed the fasting biochemical, colonic imaging, gene-expression, circulating-marker and inflammatory measurements. Complete OGTT curves and AUC values were available for five mice per group because complete serial blood samples were unavailable for the remaining main-cohort animals. OGTT and fasting glucose/insulin assessments were measured on different days. Main-cohort MOD and PF40 results are shown in the main figures. No formal a priori sample-size calculation was performed for this exploratory study.

#### 2.2.2. Separate FITC–Dextran Pilot Cohort

The permeability pilot comprised CON-FD, MOD-FD and PF40-FD mice (*n* = 3 per group), distinct from the main cohort. These animals received the assigned vehicle or PF40 schedule described in Section 2.3 and were reserved for the 4 kDa FITC–dextran assay in Section 2.5.

### 2.3. PF40 Treatment

PF40 was prepared and quality-controlled as previously reported by our group [21,22]. The final PF40 yield was 19.46% relative to the starting dry root mass, and total carbohydrate and protein contents were 80.21% and 0.11%, respectively. The UV–visible spectrum showed no distinct absorption peaks at 260 or 280 nm (Figure S3). PF40 exhibited a molecular-weight distribution of 51.6–211 kDa. Monosaccharide analysis showed predominantly glucose (85.13%) and galactose (2.21%; Figure S1). Fourier-transform infrared spectroscopy showed characteristic polysaccharide absorption bands (Figure S2). Treatment began after model confirmation. PF40 mice received 400 mg/kg by oral gavage once daily for 6 weeks, at a gavage volume of 10 mL/kg. PF40 was prepared in sterile distilled water immediately before administration and mixed thoroughly. Vehicle groups received the same volume of sterile distilled water. CON remained on maintenance feed, and MOD and PF40 remained on XTHF60. The same assigned treatment schedules applied to the main and permeability pilot cohorts.

The 400 mg/kg/day dose was selected based on our previous PF40 study in cyclophosphamide-induced immunosuppressed mice [25]. That study evaluated 100, 200 and 400 mg/kg/day and demonstrated activity at 400 mg/kg/day.

### 2.4. Oral Glucose Tolerance Test, Serum Insulin, and HOMA-IR

An oral glucose tolerance test (OGTT) was performed at the end of treatment week 6 in the main cohort, with complete curves available for five mice per group. After a 12 h fast with free access to water, tail-tip glucose was measured at 0 min. Glucose was administered by gavage at 2.0 g/kg, and glucose was measured at 30, 60, 90 and 120 min. The 0–120 min AUC was calculated for each mouse using the trapezoidal rule. Animals resumed feeding after OGTT and continued their assigned treatment until terminal sampling. FBG and fasting serum insulin (FINS) were assessed on a different day after a 12 h fast (CON-main, *n* = 7; MOD-main, *n* = 6; PF40-main, *n* = 7). Tail-tip FBG was measured, and blood was collected from the tail vein, allowed to clot for 30 min at room temperature and centrifuged at 3000× *g* for 10 min at 4 °C. FINS was measured in technical duplicate using a mouse insulin ELISA kit (Cat. No. SEKM-0387, Solarbio, Beijing, China); duplicate values were averaged within each mouse. The homeostasis model assessment of insulin resistance (HOMA-IR) was calculated from paired FBG and FINS values from the same mouse on this fasting assessment day: FBG (mmol/L) × FINS (μIU/mL)/22.5. OGTT 0 min glucose was not used in this calculation. The separate FITC–dextran pilot animals did not undergo these tests.

### 2.5. FITC–Dextran Intestinal Permeability Assay

Whole-gut macromolecular permeability was explored in a separate pilot cohort comprising CON-FD, MOD-FD and PF40-FD animals (*n* = 3 per group). This cohort was distinct from the main analysis cohort. FITC–dextran (4000 Da; Cat. No. SJ-MD0155, Shandong Sikejie Biotechnology, Jinan, China) was administered by oral gavage at 600 mg/kg after a 12 h fast with free access to water. Mice remained fasted with water available, and blood was collected 4 h later. Serum fluorescence was measured in black 96-well plates at 485 nm excitation and 528 nm emission using a Synergy H1 microplate reader (BioTek, Winooski, VT, USA). Concentrations were derived from FITC–dextran standards prepared in blank mouse serum. Results are reported for MOD-FD and PF40-FD under the treatment labels MOD and PF40; CON-FD values are provided separately in the Supplementary Material.

### 2.6. Sample Collection and Colonic Tissue Processing

After completion of the assigned tests, mice were deeply anesthetized with inhaled isoflurane. Loss of the toe-pinch reflex was confirmed before terminal blood collection from the abdominal aorta. Mice were then euthanized according to the approved animal ethics protocol, and death was confirmed. The colon was isolated immediately. From the anus toward the proximal colon, two adjacent distal colon segments containing intact fecal pellets were collected at anatomically consistent positions and ligated at both ends of each pellet. One segment was placed in prechilled Carnoy’s fixative (Cat. No. G1120, Servicebio, Wuhan, China), and the other was placed in in situ hybridization fixative (Servicebio, Cat. No. G1113). Carnoy-fixed samples were used for mucus morphology and immunofluorescence analyses. Samples in in situ hybridization fixative were used for bacterial spatial localization. Segments were neither compressed nor flushed during collection. Blood on the serosal surface was removed gently with sterile gauze to preserve the original luminal contents, mucus layer, and bacterial distribution.

The remaining colonic tissue was snap-frozen in liquid nitrogen and stored at −80 °C for ribonucleic acid (RNA) extraction, bicinchoninic acid (BCA) protein quantification, and inflammatory mediator assays. Aliquots of serum were stored at −80 °C for LPS, DAO, and D-lactate measurements. Tissue collection positions for each application were kept consistent across all animals.

### 2.7. AB/PAS Staining and Mucus Layer Morphometry

Carnoy-fixed distal colon tissue was dehydrated, cleared, infiltrated with paraffin, and embedded using standard procedures while preserving luminal contents and the luminal mucus structure. Sections were cut at 4 μm, baked, deparaffinized in xylene, and rehydrated through graded ethanol. Alcian blue/periodic acid–Schiff (AB/PAS) staining was performed with a commercial kit according to the manufacturer’s instructions. Sections underwent Alcian blue staining, periodic acid oxidation, Schiff’s development, and hematoxylin counterstaining before dehydration, clearing, and mounting.

Whole-slide images were acquired using an LG-FS80 scanner (Servicebio, Wuhan, China) with identical magnification and acquisition settings across groups. For each mouse, three nonadjacent sections spaced at least 100 μm apart were analyzed. From each section, five structurally intact, consistently oriented, nonoverlapping mucosal regions were selected by systematic equidistant sampling. Folded areas, tangential sections, epithelial detachments, and mechanically depleted mucus regions were excluded. Quantification was performed using ImageJ version 1.54q. In each region, 30 equidistant positions were selected along the luminal epithelial surface. At each position, mucus band thickness was measured perpendicular to the epithelial surface, from that surface to the outer edge of the continuous AB/PAS-positive luminal mucus band. A prespecified color threshold was applied uniformly to calculate the AB/PAS-positive area fraction within the mucosal region of interest (ROI). Measurements were averaged first across regions and sections within each animal, and each mouse was treated as an independent biological replicate. AB/PAS image analysis was performed with investigators blinded to group allocation.

### 2.8. Immunofluorescence Staining and Quantification

Paraffin-embedded colonic sections were deparaffinized and rehydrated. Heat-induced antigen retrieval was performed in Tris–ethylenediaminetetraacetic acid (EDTA) buffer (pH 9.0; Servicebio, Cat. No. G1203) at 98 °C for 20 min. After natural cooling, sections were washed with PBS (Servicebio, Cat. No. G0002) and blocked with 3% bovine serum albumin (Servicebio, Cat. No. GC305010) for 30 min at room temperature. Sections were incubated overnight at 4 °C with rabbit anti-MUC2 polyclonal antibody (Servicebio, Cat. No. GB11344; 1:500). After washing, Alexa Fluor 488-conjugated goat anti-rabbit immunoglobulin G (IgG; heavy and light chains [H + L]) (Servicebio, Cat. No. GB25303; 1:500) was applied for 50 min at room temperature in the dark. Nuclei were counterstained with 4′,6-diamidino-2-phenylindole (DAPI) (Servicebio, Cat. No. G1012) for 10 min. Sections were treated with an autofluorescence quencher (Servicebio, Cat. No. G1221) for 5 min and mounted with antifade mounting medium (Servicebio, Cat. No. G1401). Each batch included a secondary-antibody-only negative control in which the primary antibody was omitted. The suitability of the MUC2 antibody for paraffin-embedded mouse colon was supported by manufacturer validation data, the previous literature, and preliminary localization consistent with goblet cells and luminal mucus. No appreciable specific signal was observed in the negative controls.

For dual tight-junction staining, deparaffinized and rehydrated colonic sections underwent heat-induced antigen retrieval and were blocked with 5% bovine serum albumin for 1 h at room temperature. Sections were incubated overnight at 4 °C with rabbit anti-ZO-1 antibody (Servicebio, Cat. No. GB155686-100; 1:1000) and mouse anti-occludin antibody (Servicebio, Cat. No. GB15149; 1:100). After PBS washing, sections were incubated for 1 h at room temperature in the dark with cross-adsorbed Alexa Fluor 594 goat anti-rabbit IgG (H + L) (Invitrogen, Thermo Fisher Scientific, Waltham, MA, USA; Cat. No. A-11012; 1:500) and cross-adsorbed Alexa Fluor 488 goat anti-mouse IgG (H + L) (Invitrogen, Thermo Fisher Scientific, Waltham, MA, USA; Cat. No. A-11001; 1:500). ZO-1 was visualized in red and occludin in green. Nuclei were counterstained with DAPI before mounting. Each batch included secondary-antibody-only negative controls. Dual-labeling experiments also included single-label ZO-1 and occludin controls to exclude secondary-antibody cross-reactivity and fluorescence channel bleed-through. Antibody suitability in paraffin-embedded mouse colon was supported by manufacturer validation, the previous literature, and preliminary localization consistent with epithelial junctional regions.

Images were acquired using an LG-FS80 whole-slide scanner (Servicebio). Objective magnification, light intensity, exposure time, and gain were held constant across groups for each target. For each target in each mouse, three nonadjacent sections spaced at least 100 μm apart were analyzed. From each section, three to five structurally intact, nonoverlapping mucosal fields were sampled systematically. Folded areas, tangential sections, epithelial detachment, and regions with marked autofluorescence were excluded. ImageJ version 1.54q was used to subtract the background uniformly and apply a fixed, prespecified threshold. MUC2-, ZO-1-, and occludin-positive areas were calculated as percentages of the mucosal ROI. Measurements were averaged first across fields and sections within each animal, and each mouse was treated as an independent biological replicate. Image analysis was performed with investigators blinded to group allocation.

### 2.9. Reverse-Transcription Quantitative Polymerase Chain Reaction (qPCR) of Mucus-Related Genes

Frozen colonic tissue was thoroughly pulverized under liquid nitrogen, and total RNA was extracted using TRIzol™ Reagent (Invitrogen, Thermo Fisher Scientific, Waltham, MA, USA; Cat. No. 15596026). RNA concentration and purity were measured with a microvolume spectrophotometer. RNA with an A260/A280 ratio of 1.8–2.0 and acceptable quality was used for subsequent experiments. Equal amounts of total RNA were reverse-transcribed into complementary DNA (cDNA) using a High-Capacity cDNA Reverse Transcription Kit (Applied Biosystems, Thermo Fisher Scientific, Waltham, MA, USA; Cat. No. 4368814), according to the manufacturer’s instructions. qPCR was performed using PowerUp™ SYBR™ Green Master Mix (Applied Biosystems, Thermo Fisher Scientific, Waltham, MA, USA; Cat. No. A25742) and a QuantStudio™ 5 Real-Time PCR System (Applied Biosystems, Thermo Fisher Scientific, Waltham, MA, USA). The amplification program comprised 2 min at 50 °C and 2 min at 95 °C, followed by 40 cycles of 15 s at 95 °C and 1 min at 60 °C. Melt curve analysis was conducted after amplification. Each sample was assayed using two technical replicates with a no-template control. *Actb* and *Rplp0* served as dual reference genes. After confirming their stability across groups, the geometric mean of their relative expression values was used as the normalization factor. Relative *Tff3*, *Clca1*, *Fcgbp*, and *Agr2* expression was calculated using the 2^−ΔΔCt^ method in accordance with established reporting principles. All primer pairs produced a single melt peak. Amplification efficiencies ranged from 97.3% to 100.2%, and all standard curve R^2^ values exceeded 0.999. Primer sequences are listed in Table 1.

### 2.10. Fluorescence In Situ Hybridization for Bacterial Spatial Localization

Fluorescence in situ hybridization (FISH) targeting bacterial 16S ribosomal RNA (rRNA) was used to visualize the spatial distribution of bacterial signals in colonic tissue and their position relative to the epithelial surface [26,27]. Colon samples fixed in in situ hybridization fixative and embedded in paraffin were cut into 4 μm sections. After deparaffinization and rehydration, sections were equilibrated in preheated hybridization buffer and incubated with the universal bacterial probe EUB338 labeled with FITC at the 5′ end. The EUB338 sequence was 5′-GCTGCCTCCCGTAGGAGT-3′ and was custom synthesized by Tsingke Biotechnology Co., Ltd. (Beijing, China).

The hybridization buffer contained 0.9 mol/L sodium chloride (NaCl), 20 mmol/L Tris–hydrochloride (Tris–HCl), 0.01% sodium dodecyl sulfate (SDS), and 30% formamide at pH 7.2. The final EUB338–FITC probe concentration was 5 ng/μL. Each section received 100 μL of probe-containing hybridization solution and was incubated for 16 h at 45 °C in a humidified chamber protected from light. Sections were then washed for 20 min in wash solution preheated to 48 °C, rinsed with ribonuclease-free PBS, counterstained with DAPI, and mounted in antifade medium.

To evaluate nonspecific probe binding and tissue background, adjacent sections were hybridized in parallel with the FITC-labeled nonsense negative-control probe NON338. The NON338 sequence was 5′-ACTCCTACGGGAGGCAGC-3′. Probe concentration, hybridization buffer composition, hybridization temperature and duration, washing conditions, counterstaining, and mounting were identical to those used for EUB338–FITC. EUB338 and NON338 sections were imaged using identical objective magnification, light intensity, exposure time, gain, and fluorescence display range. No appreciable specific fluorescence was observed in NON338 sections.

Images were acquired using an LG-FS80 whole-slide scanner (Servicebio) with identical objective magnification, light intensity, exposure time, and gain for EUB338 sections and their adjacent NON338 controls. For each mouse, three nonadjacent EUB338 sections spaced at least 100 μm apart were analyzed. From each section, three to five structurally intact, nonoverlapping fields were sampled systematically. Folded areas, tangential sections, epithelial detachment, and regions with marked autofluorescence were excluded. A uniform ImageJ threshold was used to identify EUB338-positive signals. Investigators traced the epithelial-facing boundary of EUB338-positive signals and the luminal boundary of the colonic epithelium. At least 20 equidistant points were selected along the luminal epithelial boundary in each field. The shortest Euclidean distance from each point to the epithelial-facing EUB338-positive signal boundary was measured. Pointwise distances were averaged within fields, sections and individual animals, and each mouse was treated as an independent biological replicate. Image analysis was performed with investigators blinded to group allocation. The two dashed lines in in the representative FISH images denote the EUB338-positive signal boundary and the luminal epithelial boundary used for distance measurements.

EUB338-positive distance is a two-dimensional descriptor of bacterial rRNA-associated signal localization. It does not identify bacterial taxonomy, viability, absolute abundance, microbial function or mucin-degrading capacity, and does not directly measure mucus penetrability or functional barrier integrity.

### 2.11. Serum LPS, DAO, and D-Lactate Measurements

Frozen serum was thawed slowly on ice, mixed gently, and centrifuged to remove precipitates. Repeated freeze–thaw cycles were avoided. Serum immunoreactive lipopolysaccharide (LPS) was measured using a mouse LPS ELISA kit (Shanghai Enzyme-linked Biotechnology Co., Ltd., Shanghai, China; Cat. No. ml503352) with pyrogen-free consumables. Serum diamine oxidase (DAO) was measured using a mouse DAO ELISA kit (Shanghai Enzyme-linked Biotechnology Co., Ltd., Shanghai, China; Cat. No. ml002199). Both ELISAs were performed according to the manufacturer’s instructions. Concentrations were calculated from standard curves, and samples outside the linear range were diluted appropriately and reassayed. Serum D-lactate was measured using a colorimetric D-lactate assay kit (Nanjing Jiancheng Bioengineering Institute, Nanjing, China; Cat. No. A019-3-1). Samples were processed and developed according to the manufacturer’s instructions. Absorbance was measured at 450 nm, and D-lactate concentrations were calculated from the standard curve.

### 2.12. Inflammatory Mediators in Colonic Tissue Homogenates

Frozen colonic tissue was combined with prechilled PBS and protease inhibitor at a tissue mass (g)-to-buffer volume (mL) ratio of 1:9. Samples were homogenized thoroughly on ice to prepare 10% (*w*/*v*) tissue homogenates. Homogenates were centrifuged at 12,000× *g* for 10 min at 4 °C. Supernatants were collected, aliquoted, and stored without repeated freeze–thaw cycles. Total protein concentration was measured using a bicinchoninic acid (BCA) assay kit (Nanjing Jiancheng Bioengineering Institute, Nanjing, China; Cat. No. A045-3). Before measurement, samples were diluted 1:9 to keep the absorbance within the linear range of the standard curve. Absorbance was read at 562 nm, and protein concentration was calculated from a bovine serum albumin standard curve. Mouse C-C motif chemokine ligand 2/monocyte chemoattractant protein-1 (CCL2/MCP-1), TNF-α, IL-1β, and IL-6 ELISA kits (MultiSciences (Lianke) Biotech Co., Ltd., Hangzhou, China; Cat. Nos. EK287, EK282, EK201B, and EK206, respectively) were used to measure the corresponding mediators in homogenate supernatants. Concentrations were calculated from standard curves and normalized to total protein concentration.

### 2.13. Statistical Analysis

Statistical analyses were performed using WPS Office version 12.1.0.26895 (Kingsoft Office Software, Beijing, China). Individual mice were independent biological replicates; imaging fields, sections and technical replicates were averaged within each animal. Inferential comparisons were restricted to MOD versus PF40. Most continuous endpoints were compared using two-sided Welch unpaired *t* tests. Exact two-sided Mann–Whitney rank permutation tests were used for OGTT AUC, occludin-positive area and the small FITC–dextran pilot because of sample-size or distributional concerns. All group-label allocations were enumerated, with average ranks for ties. Complementary Welch or exact rank tests assessed sensitivity to the method. A finding is termed method-sensitive when the same data yield *p* values on opposite sides of the nominal 0.05 threshold under the two tests; these findings are reported in the Results. OGTT curves are descriptive mean ± SEM; other figure error bars and supplementary summaries are mean ± SD. Baseline values are descriptive. Body weight was followed in all allocated animals (CON, *n* = 10; MOD, *n* = 9; PF40, *n* = 10); final weight gain, calculated as study week 12 minus study week 6 for each mouse, was compared between MOD and PF40 using a two-sided Welch test. Weekly measurements were not treated as independent animals. All endpoint tests are exploratory, including analyses added during revision. Nominal *p* < 0.05 was used without adjustment across endpoints; the resulting observations require confirmation in studies designed to account for multiplicity.

## 3. Results

### 3.1. Fasting Metabolic Measures After PF40 Treatment in the Shared XTHF60/STZ Background

**Figure 1:**
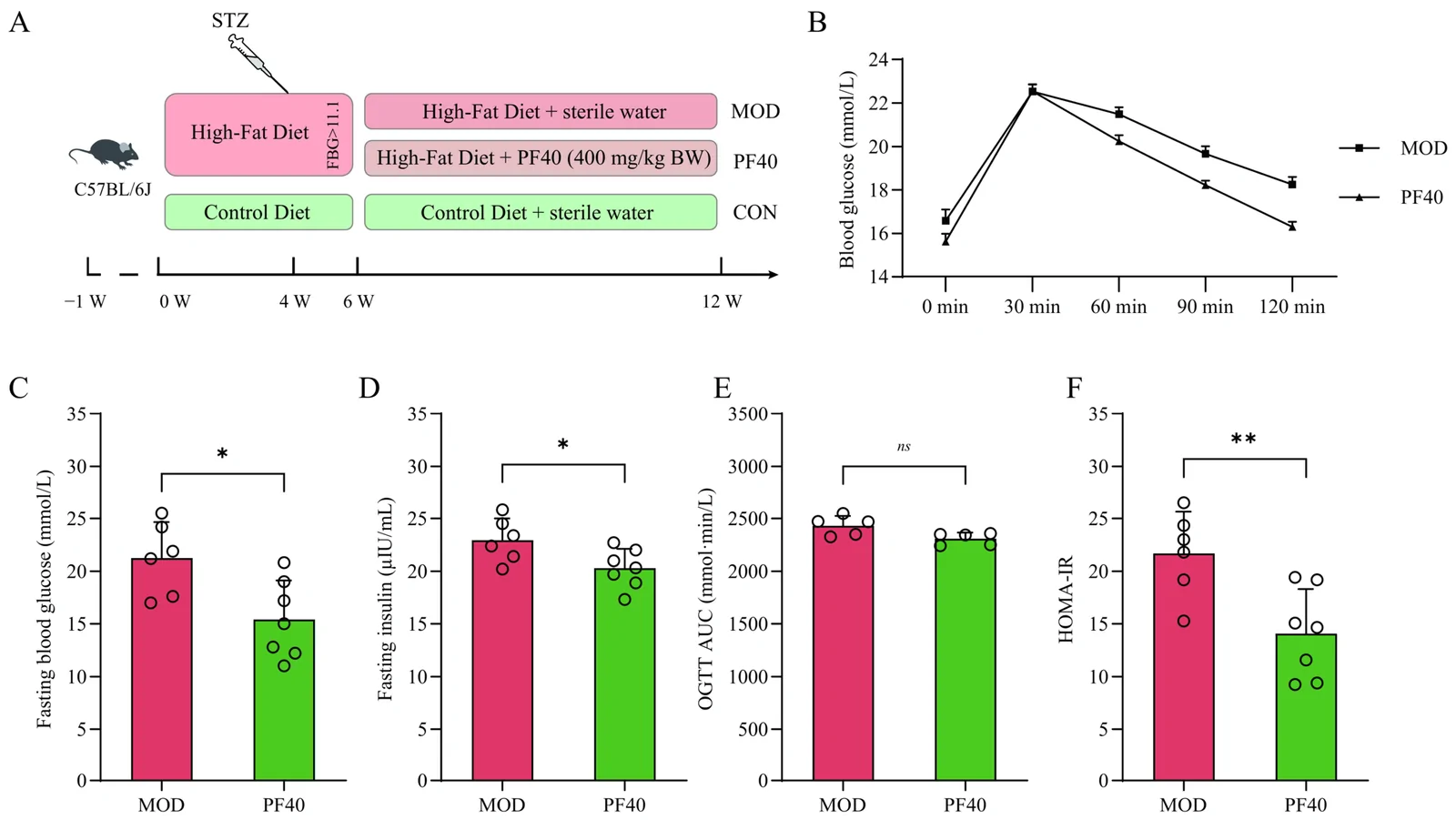
Fasting metabolic measures and descriptive OGTT curves in the XTHF60/STZ model. (**A**) Model-induction and treatment timeline, including the separately reported reference cohort. (**B**) OGTT glucose curve, mean ± SEM. (**C**) Fasting blood glucose. (**D**) Fasting insulin. (**E**) OGTT AUC. (**F**) HOMA-IR calculated from same-animal FBG/FINS on the fasting biochemical assessment day. Panels B and E: *n* = 5 mice per treatment group. Panels (**C**,**D**,**F**): MOD-main, *n* = 6; PF40-main, *n* = 7. The smaller OGTT sample reflects incomplete serial sampling; OGTT and FBG/FINS were measured on different days. Insulin technical duplicates were averaged within animals. Each dot in (**C**–**F**) represents one mouse; bars show mean ± SD. Brackets compare MOD and PF40 only. Tests: (**C**,**D**,**F**), two-sided Welch unpaired *t* tests; (**E**), exact two-sided Mann–Whitney rank permutation test. * *p* < 0.05; ** *p* < 0.01; ns, not significant. The Welch sensitivity result for (**E**) was *p* = 0.0410, whereas the primary rank result was *p* = 0.1111; the displayed ns refers to the primary test. CON outcomes are reported separately in Table S1.

Mice received PF40 or vehicle for 6 weeks after model induction (Figure 1A). PF40 mice had lower FBG (Welch *p* = 0.0136), FINS (*p* = 0.0336) and HOMA-IR (*p* = 0.0068) than MOD (Figure 1C,D,F). FINS was method-sensitive: the exact rank sensitivity result was *p* = 0.0513. OGTT AUC was numerically lower with PF40 but nonsignificant in the primary exact rank test (*p* = 0.1111; Figure 1E). The Welch sensitivity result was *p* = 0.0410. CON values are presented separately in Table S1.

Post-randomization pretreatment FBG was 15.18 ± 1.76 mmol/L in MOD and 15.32 ± 1.51 mmol/L in PF40; body weight was 27.80 ± 1.38 and 27.69 ± 1.66 g, respectively (mean ± SD; *n* = 9 and 10; Table S5). Three-group weekly weights are summarized in Table S6. Final body-weight gain was 1.45 ± 0.49 g in MOD and 1.64 ± 0.59 g in PF40 (Welch *p* = 0.4539).

### 3.2. PF40 Is Associated with a Thicker Continuous Colonic Mucus Band than Vehicle

**Figure 2:**
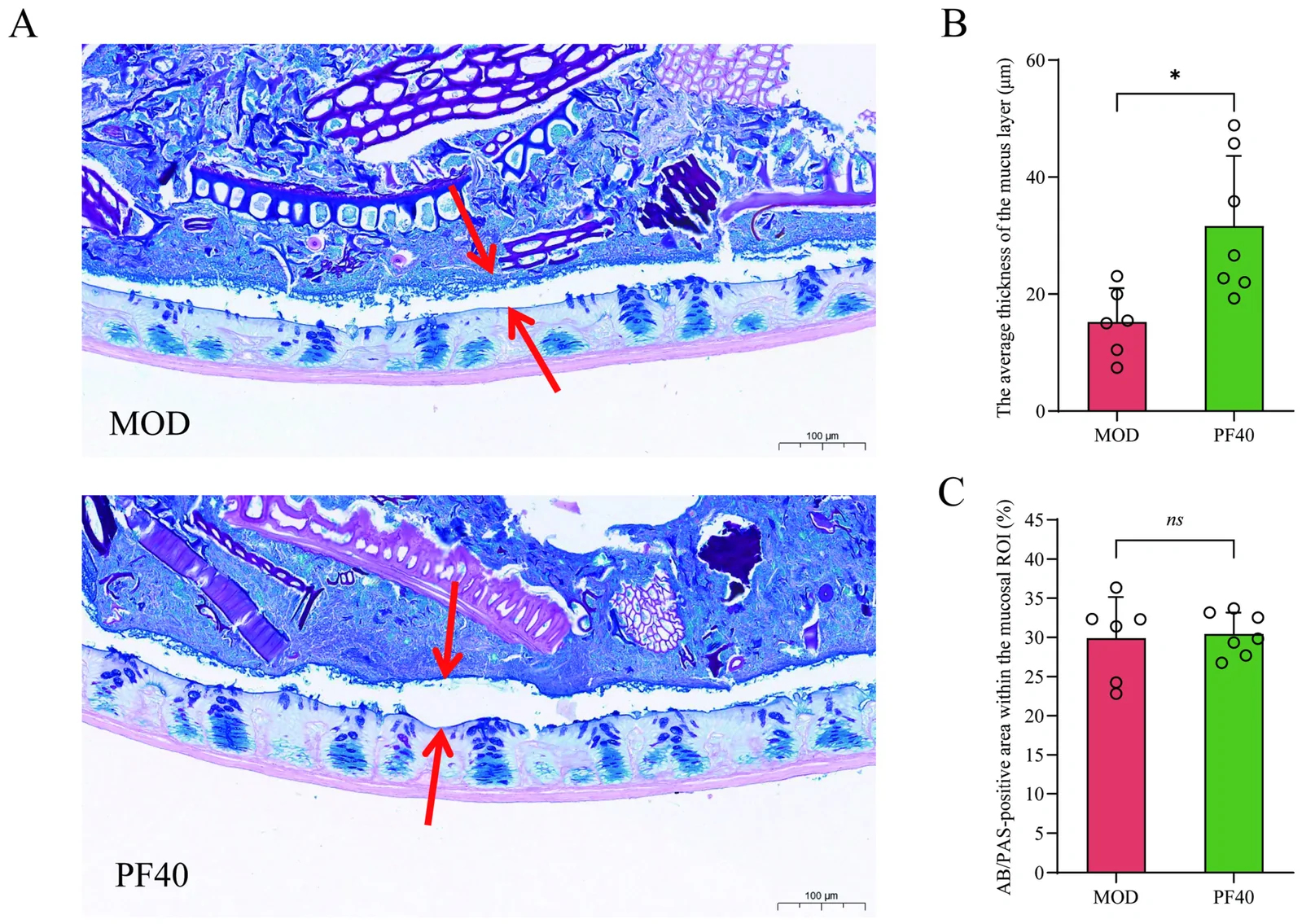
Continuous AB/PAS-positive luminal mucus band in MOD and PF40 mice. (**A**) Representative AB/PAS-stained colonic sections; red arrows identify the retained luminal mucus band. Scale bars, 100 μm. (**B**) Mean band thickness. (**C**) AB/PAS-positive area fraction within the mucosal ROI. MOD-main, *n* = 6; PF40-main, *n* = 7. Section and field measurements were averaged within each animal; each dot represents one mouse. Bars show mean ± SD. Brackets compare MOD and PF40 using two-sided Welch unpaired *t* tests. * *p* < 0.05; ns, not significant. CON images and descriptive values are reported separately in Figure S4A and Table S2.

PF40 mice had a thicker continuous AB/PAS-positive luminal mucus band than MOD (Welch *p* = 0.0110), with greater apparent continuity in the representative sections (Figure 2). The mucosal AB/PAS-positive area fraction did not differ significantly (*p* = 0.8323). CON images and descriptive values are provided separately in Figure S4A and Table S2.

### 3.3. Colonic MUC2-Positive Area and Mucus-Related Genes in the HFD/STZ Model

**Figure 3:**
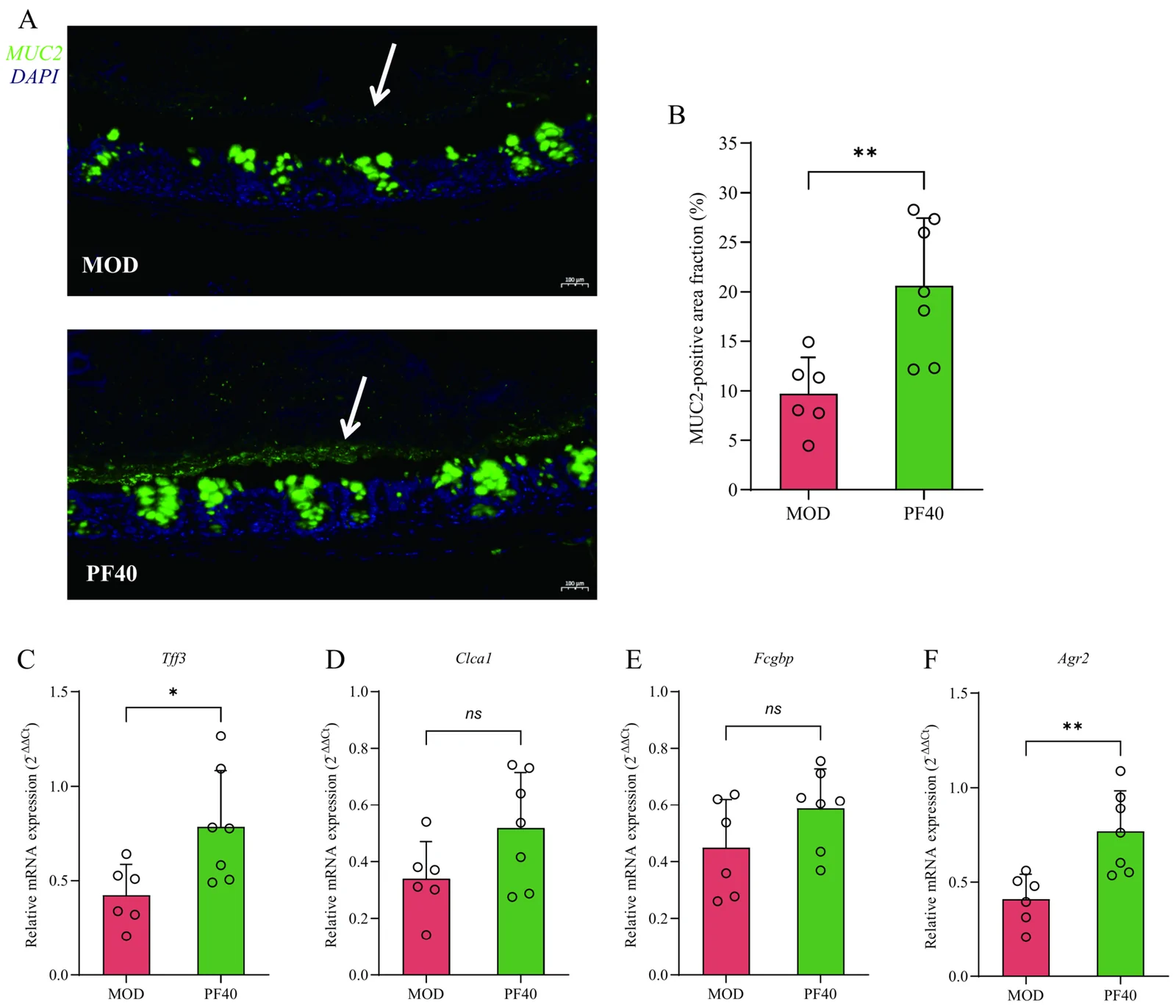
MUC2-positive area and mucus-related gene expression in MOD and PF40 mice. (**A**) Representative colonic MUC2 immunofluorescence; MUC2 is green and DAPI-stained nuclei are blue. White arrows indicate the retained luminal MUC2-positive mucus band. Scale bars, 100 μm. (**B**) MUC2-positive area fraction. (**C**–**F**) Relative *Tff3*, *Clca1*, *Fcgbp* and *Agr2* mRNA expression. MOD-main, *n* = 6; PF40-main, *n* = 7. Imaging measurements and two qPCR technical replicates were averaged within each animal. Each dot represents one mouse; bars show mean ± SD. Brackets compare MOD and PF40 using two-sided Welch unpaired *t* tests. * *p* < 0.05; ** *p* < 0.01; ns, not significant. For *Tff3*, Welch *p* = 0.0210 and exact rank sensitivity *p* = 0.0513. CON images and descriptive values are reported separately in Figure S4B and Table S2.

Relative to MOD, PF40 mice had a larger MUC2-positive area fraction (Welch *p* = 0.0049), higher *Tff3* expression (*p* = 0.0210) and higher *Agr2* expression (*p* = 0.0043; Figure 3). The *Tff3* result was method-sensitive (exact rank sensitivity *p* = 0.0513). *Clca1* (Welch *p* = 0.0797) and *Fcgbp* (*p* = 0.1438) did not differ significantly. CON images and descriptive values are provided separately in Figure S4B and Table S2.

### 3.4. PF40 Is Associated with Greater Bacterial–Epithelial Distance than Vehicle

**Figure 4:**
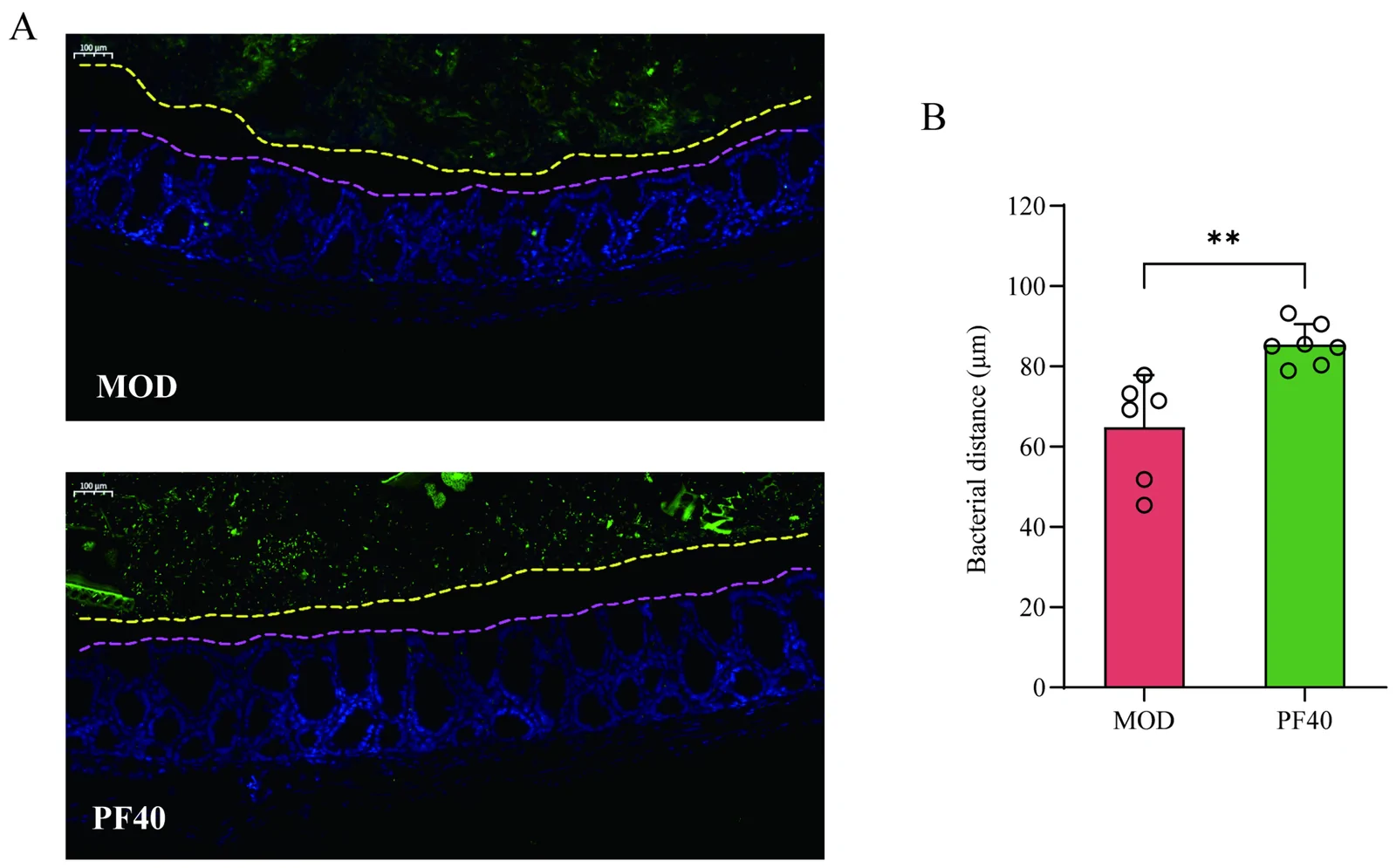
EUB338-positive signal–epithelial distance in MOD and PF40 mice. (**A**) Representative FISH images; FITC-labeled EUB338 signals are green and DAPI-stained nuclei are blue. Dashed lines identify the epithelial-facing signal boundary and luminal epithelial boundary used for the two-dimensional distance measurement. Scale bars, 100 μm. (**B**) Mean distance from sampled epithelial points to the EUB338-positive signal boundary, calculated from pointwise shortest Euclidean distances. MOD-main, *n* = 6; PF40-main, *n* = 7. Section and field measurements were averaged within each animal; each dot represents one mouse. Bars show mean ± SD. The bracket compares MOD and PF40 using a two-sided Welch unpaired *t* test. ** *p* < 0.01. CON images and values are reported separately in Figure S4C and Table S2.

EUB338-FISH showed a greater animal-averaged distance from sampled epithelial points to the EUB338-positive signal boundary in PF40 than MOD (Welch *p* = 0.0098; Figure 4). CON images and descriptive distances are reported separately in Figure S4C and Table S2.

### 3.5. Circulating Barrier-Related Measures and Pilot FITC–Dextran Permeability

**Figure 5:**
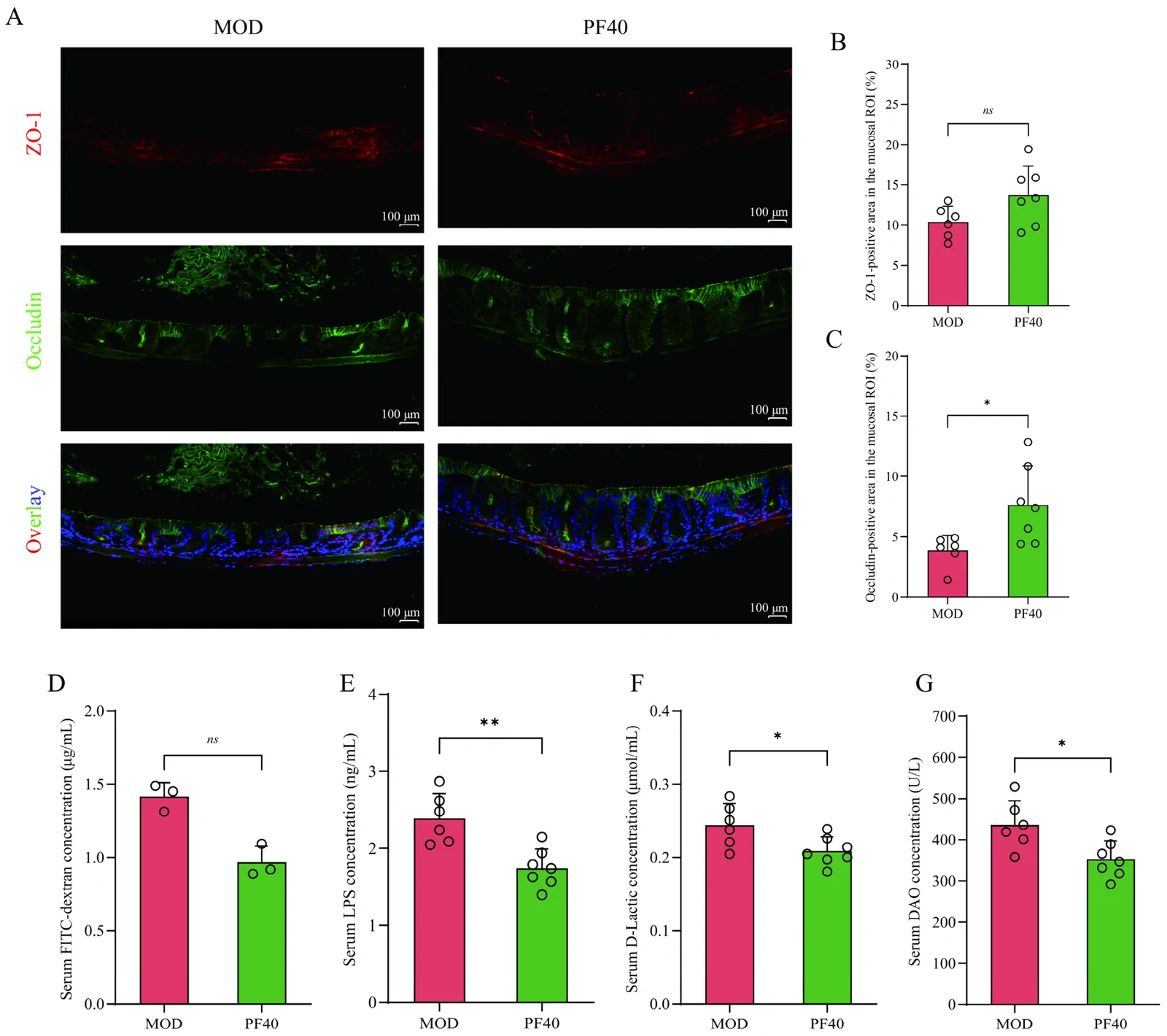
Main-cohort barrier-related measures and a separate FITC–dextran pilot. (**A**) Colonic dual immunofluorescence; ZO-1 is red, occludin is green and DAPI-stained nuclei are blue. Scale bars, 100 μm. (**B**,**C**) ZO-1- and occludin-positive area fractions. (**D**) Serum FITC–dextran 4 h after oral 4 kDa FITC–dextran, from separate MOD-FD/PF40-FD pilot animals (*n* = 3 per arm), shown under treatment labels MOD/PF40. These animals did not contribute to (**B**,**C**,**E**–**G**). (**E**–**G**) Serum immunoreactive LPS, D-lactate and DAO. Main-cohort panels: MOD-main, *n* = 6; PF40-main, *n* = 7. Imaging measurements were averaged within each animal. Each dot represents one mouse; bars show mean ± SD. Tests: (**B**,**E**–**G**), two-sided Welch unpaired *t* tests; (**C**,**D**), exact two-sided Mann–Whitney rank permutation tests. * *p* < 0.05; ** *p* < 0.01; ns, not significant. For (**D**), primary exact *p* = 0.1000 and Welch sensitivity *p* = 0.0060; ns refers to the primary test. CON data are presented separately in Figure S5 and Table S3.

In the main cohort, PF40 mice had lower serum LPS (Welch *p* = 0.0029), D-lactate (*p* = 0.0333) and DAO (*p* = 0.0192) than MOD. Occludin-positive area was higher in the primary exact rank test (*p* = 0.0140). ZO-1-positive area did not differ significantly (Welch *p* = 0.0631; Figure 5A–C,E–G). The separate FITC–dextran pilot (MOD-FD/PF40-FD, *n* = 3 per arm) showed lower mean serum concentrations with PF40 (Figure 5D). The primary exact rank result was *p* = 0.1000; the Welch sensitivity result was *p* = 0.0060. Given the small sample and method sensitivity, this pilot is inconclusive and requires larger-cohort validation. CON-main and CON-FD data are provided separately in Figure S5 and Table S3.

### 3.6. PF40 Reduces Selected Colonic Proinflammatory Mediator Levels

**Figure 6:**
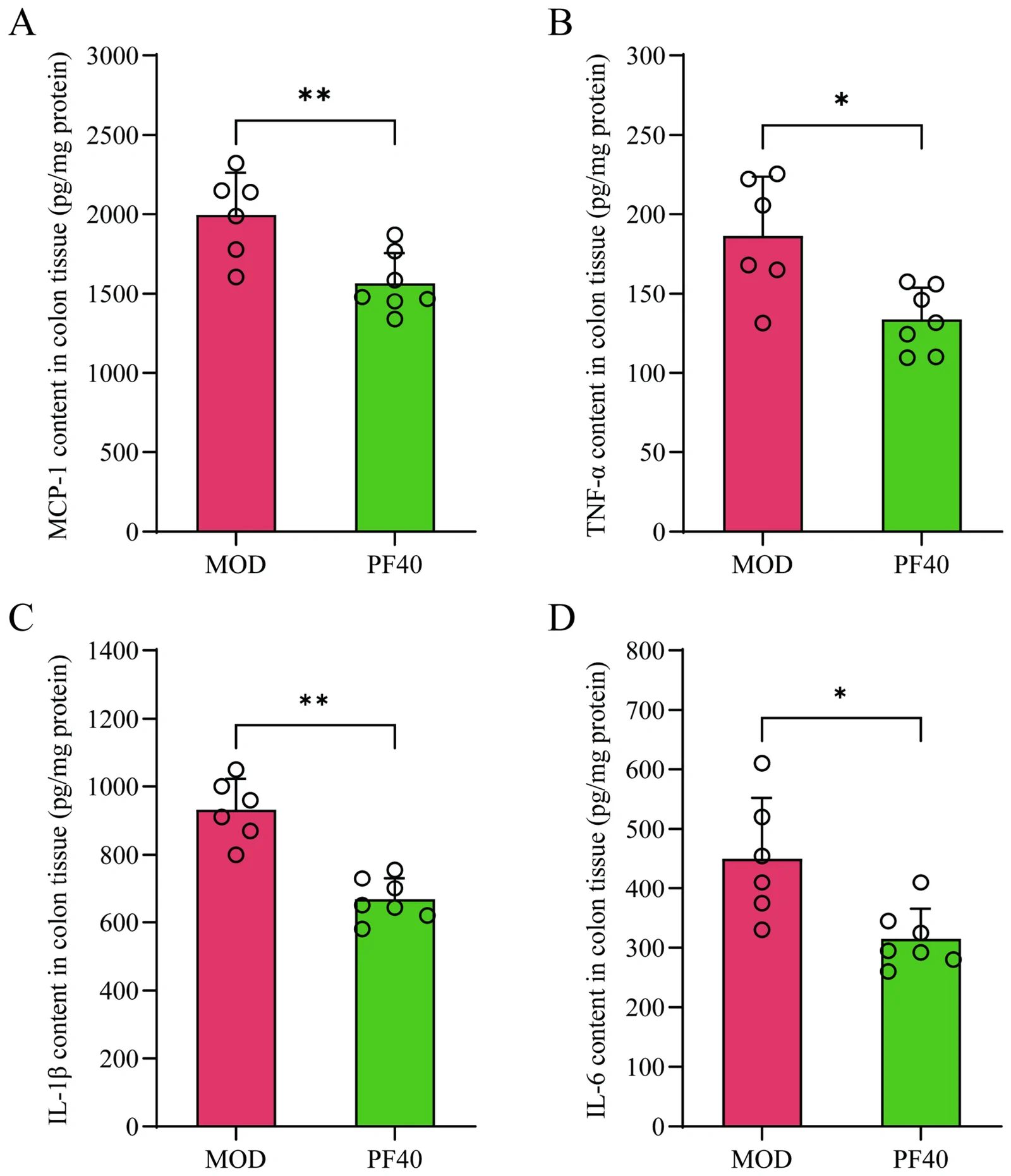
Colonic inflammatory mediator levels in MOD and PF40 mice. (**A**–**D**) MCP-1, TNF-α, IL-1β and IL-6, normalized to total tissue protein (pg/mg protein). MOD-main, *n* = 6; PF40-main, *n* = 7. Each dot represents one mouse; bars show mean ± SD. Brackets compare MOD and PF40 using two-sided Welch unpaired *t* tests. * *p* < 0.05; ** *p* < 0.01. CON descriptive values are presented separately in Table S4.

PF40 mice had lower colonic tissue MCP-1 (Welch *p* = 0.0088), TNF-α (*p* = 0.0164), IL-1β (*p* = 0.000232) and IL-6 (*p* = 0.0216) than MOD (Figure 6). CON values are presented separately in Table S4.

## 4. Discussion

PF40-treated mice showed a thicker retained colonic mucus band, larger MUC2-positive area and greater bacterial–epithelial distance than vehicle-treated mice. These differences occurred under shared XTHF60/STZ conditions, alongside lower circulating barrier-related markers, colonic inflammatory mediators and fasting metabolic measures. Combining mucus morphology with bacterial spatial imaging characterizes complementary intestinal phenotypes associated with PF40 treatment in this model.

### 4.1. Colonic Mucus and Bacterial Spatial Findings Within the Shared Dietary Background

The greater retained mucus band thickness and MUC2-positive area in PF40-treated mice characterize differences in the colonic mucus phenotype. The colonic mucus layer consists primarily of gel-forming mucins secreted by goblet cells. MUC2 polymerization and extensive glycosylation yield a hydrated gel with regions of differing density. The dense inner layer limits bacterial access, whereas the outer layer provides a habitat and nutrients for some microorganisms [6,7,8,10]. Metabolic abnormalities have been associated with slower mucus growth, increased penetrability and microbial encroachment, and shorter bacterial–epithelial distance is associated with dysglycemia in humans [16,17,18]. These findings provide biological context for examining mucus morphology and spatial measurements together.

Higher *Agr2* expression provides a biological link for investigating mucus production and maturation. MUC2 synthesis, folding, polymerization and exocytosis contribute to mucus formation [7,9]. AGR2 supports MUC2 folding and maturation, and AGR2 loss impairs mucus production [28]. The concurrent mucus and *Agr2* findings therefore identify mucin production and maturation as candidate processes for further study. TFF3 contributes to epithelial protection and repair [29]; its expression result here depended on the statistical test. *Clca1*, *Fcgbp* and overall mucosal AB/PAS-positive area did not differ significantly. AB/PAS-positive area includes intracellular and luminal material, and goblet-cell number was not quantified.

The greater EUB338-positive signal–epithelial distance adds a spatial dimension to the histological findings. Imaging of bacterial proximity complements mucus measurements [16,26,27,30]. Spatial organization may depend on mucus density, glycosylation, renewal, antimicrobial distributions, adhesion and mucin utilization [7,8,11,12]. These two-dimensional EUB338 rRNA-signal distances depend on section geometry and thresholds; bacterial taxonomy, viability, absolute abundance, function, mucin degradation, mucus penetrability and functional integrity require separate assays.

### 4.2. Concurrent Barrier-Related and Colonic Inflammatory Findings

The intestinal barrier comprises mucus, epithelial cells and junctions, antimicrobial molecules and mucosal immunity. Literature evidence connects hyperglycemia with epithelial transcriptional changes and junctional dysfunction [3], and tight-junction regulation with permeability and inflammation [31]. Circulating markers, junctional fluorescence coverage and colonic mediators characterize complementary features of the intestinal phenotype.

PF40-treated mice showed greater occludin-positive fluorescence coverage, whereas ZO-1-positive area did not differ significantly between groups. The increased occludin coverage provides evidence of a junction-associated histological response. Positive-area fraction describes thresholded fluorescence coverage within the mucosal ROI. Direct measurements of total protein abundance, tight-junction organization and epithelial barrier function are needed to assess the biological and functional significance of the increased coverage [31,32].

Lower serum LPS, D-lactate and DAO provide complementary circulating observations alongside the colonic imaging findings [33]. Circulating LPS also has inflammatory and metabolic relevance [5]. These markers do not localize the anatomical source of the change.

Intestinal permeability and mucosal inflammation can influence one another through microbial products, inflammatory signaling and junctional regulation [4,8,31]. Lower colonic MCP-1, TNF-α, IL-1β and IL-6 place the tissue inflammatory findings alongside the mucus, spatial and circulating-marker phenotypes. This combination motivates studies that track local inflammatory signaling together with mucus dynamics and bacterial localization.

### 4.3. Interpretation of Concurrent Metabolic and Barrier-Related Findings

Lower fasting glucose and HOMA-IR accompany the colonic findings. Fasting insulin and OGTT AUC showed test-dependent statistical results (Section 3.1), so the metabolic interpretation centers on exploratory fasting parameters. Previous PF40 studies reported metabolic and intestinal effects in other animal settings [21,22], providing context for follow-up of the intestinal and metabolic observations.

PF40-associated differences in mucus, bacterial localization, barrier-related measures, inflammation and fasting metabolism were measured at the study endpoint. Concurrent endpoint measurements do not resolve the temporal order or causal mediation linking mucus, bacterial localization, barrier function, inflammation and glucose regulation. Lower glycemic exposure could also influence intestinal phenotypes [3]. Longitudinal sampling and interventions that separately manipulate mucus, microbial localization or glycemia would help determine direction, timing and mediation.

### 4.4. Limitations and Translational Considerations

The study evaluated one PF40 dose for 6 weeks in male C57BL/6J mice and included no pharmacological positive control. Sample sizes were selected without prospective power calculations. Unadjusted testing across multiple endpoints increases the risk of false-positive findings, and the observed differences remain exploratory. The separate FITC–dextran pilot (*n* = 3 per arm) was inconclusive and requires validation in a larger, prospectively sized cohort. Studies including both sexes, dose-ranging, longer follow-up and an established treatment comparator would assess the response range, durability and relative efficacy.

CON received grain-based maintenance feed, whereas MOD and PF40 received the defined XTHF60 formulation. Differences in fiber and other dietary components may influence microbial use of mucus glycans [15]. This dietary mismatch limits comparisons with CON; the randomized MOD–PF40 comparison evaluates treatment-associated differences within the shared XTHF60/STZ setting. A compositionally matched non-diabetic control would permit assessment of recovery toward a matched healthy reference. Final body-weight gain did not differ significantly between MOD and PF40. Food intake, energy intake and nutrient absorption were not measured or recorded, so their contribution to the intestinal and fasting metabolic findings remains unresolved.

Using mouse and adult human Km factors of 3 and 37, 400 mg/kg/day corresponds to approximately 32.4 mg/kg/day [34]. For a 60 kg adult, this is approximately 1.95 g/day of PF40. At the reported 19.46% extraction yield, this corresponds to approximately 10 g/day of dried *P. heterophylla* root. This amount falls within the 9–30 g dosage range specified in the Chinese Pharmacopoeia [35]. These estimates provide dose context; clinical PF40 dosing requires dedicated exposure, safety and efficacy studies.

## 5. Conclusions

PF40-treated mice exhibited greater retained colonic mucus thickness, increased MUC2-positive area and greater bacterial–epithelial distance than vehicle-treated mice under shared XTHF60/STZ conditions. These findings were accompanied by higher occludin-positive coverage, lower circulating barrier-related markers, reduced colonic inflammatory mediators and lower fasting glucose and HOMA-IR in exploratory analyses. The results identify colonic mucus morphology and bacterial spatial localization as treatment-responsive phenotypes associated with PF40 administration in this model. The causal contribution of these intestinal changes to barrier function, inflammation and glucose regulation remains to be established and warrants direct mechanistic investigation.

## Figures and Tables

**Table 1:** qPCR primer sequences for mouse colonic tissues.

Gene	National Center for Biotechnology Information (NCBI) Reference Sequence (RefSeq) Accession	Primer Sequences (5′–3′; F, Forward; R, Reverse)
*Tff3*	NM_011575	F: TCCAAGCCAATGTATGGTGCCG
		R: CAGGGCACATTTGGGATACTGG
*Agr2*	NM_011783	F: GCAGTTTGTTCTCCTCAACCTGG
		R: GTATCGTCCAGTGATGTCTGCC
*Clca1*	NM_017474	F: CTGCCGCTAAAGAGCTTGAGCA
		R: ATCGCCGCATTTCCTGAGGAGA
*Fcgbp*	NM_001122603	F: CCATCCACAAGAATGAGATCGGC
		R: GTCAGTCTCCAGCACAGCCTTT
*Actb*	NM_007393	F: CATTGCTGACAGGATGCAGAAGG
		R: TGCTGGAAGGTGGACAGTGAGG
*Rplp0*	NM_007475.5	F: AGATTCGGGATATGCTGTTGGC
		R: TCGGGTCCTAGACCAGTGTTC

## Data Availability

The data presented in this study are available on request from the corresponding author. Descriptive endpoint, baseline and body-weight summaries are provided in the Supplementary Tables. Representative unedited colonic images are provided in Supplementary File S1.

## Supplementary Materials

The following supporting information can be downloaded at: https://www.mdpi.com/article/10.3390/cimb48101093/s1.

## Abbreviations

The following abbreviations are used in this manuscript:

T2DM	type 2 diabetes mellitus
STZ	streptozotocin
AB/PAS	Alcian blue/periodic acid–Schiff
FISH	fluorescence in situ hybridization
FBG	fasting blood glucose
FINS	fasting serum insulin
HOMA-IR	homeostasis model assessment of insulin resistance
OGTT	oral glucose tolerance test
IL-1β	interleukin-1 beta
IL-6	interleukin-6
TNF-α	tumor necrosis factor alpha
LPS	lipopolysaccharide
MCP-1	monocyte chemoattractant protein-1
DAO	diamine oxidase
EUB338	universal bacterial 16S rRNA-targeting probe EUB338
NON338	nonsense negative-control probe complementary to EUB338
ZO-1	zonula occludens-1
*T**ff3*	trefoil factor 3
*Clca1*	chloride channel accessory 1
*Fcgbp*	Fc fragment of IgG binding protein
*Agr2*	anterior gradient 2
PF40	*Pseudostellaria heterophylla* polysaccharide fraction PF40
CON	non-diet-matched reference group
MOD	model group
MUC2	mucin 2
FITC	fluorescein isothiocyanate
AUC	area under the curve
ROI	region of interest
mRNA	messenger ribonucleic acid
qPCR	quantitative polymerase chain reaction
ARRIVE	Animal Research: Reporting of In Vivo Experiments
FD&C	Food, Drug, and Cosmetic
ELISA	enzyme-linked immunosorbent assay
PBS	phosphate-buffered saline
BCA	bicinchoninic acid
EDTA	ethylenediaminetetraacetic acid
IgG	immunoglobulin G
H + L	antibody heavy and light chains
DAPI	4′,6-diamidino-2-phenylindole
RNA	ribonucleic acid
cDNA	complementary DNA
rRNA	ribosomal RNA
NaCl	sodium chloride
Tris–HCl	Tris hydrochloride
SDS	sodium dodecyl sulfate
CCL2	C-C motif chemokine ligand 2
NCBI	National Center for Biotechnology Information
RefSeq	Reference Sequence
