# Supplementary material for: Pseudostellaria heterophylla Polysaccharide Increases Colonic Mucus Thickness and Bacterial–Epithelial Distance in HFD/STZ-Induced Diabetic Mice
Source: Curr Issues Mol Biol. 2026 Oct 21;48(10):1093. doi: 10.3390/cimb48101093 (PMC13702876; doi:10.3390/cimb48101093)
Supplement: Supplementary file 1 [file cimb-48-01093-s001.zip › cimb-4607865-supplementary.pdf]

# Supplementary Materials

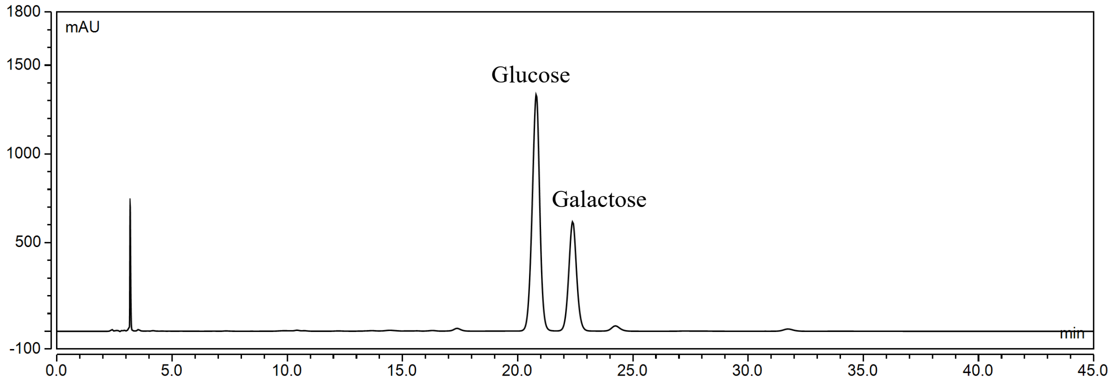


**Figure S1**. Monosaccharide composition of PF40. PF40 was mainly composed of glucose and galactose.

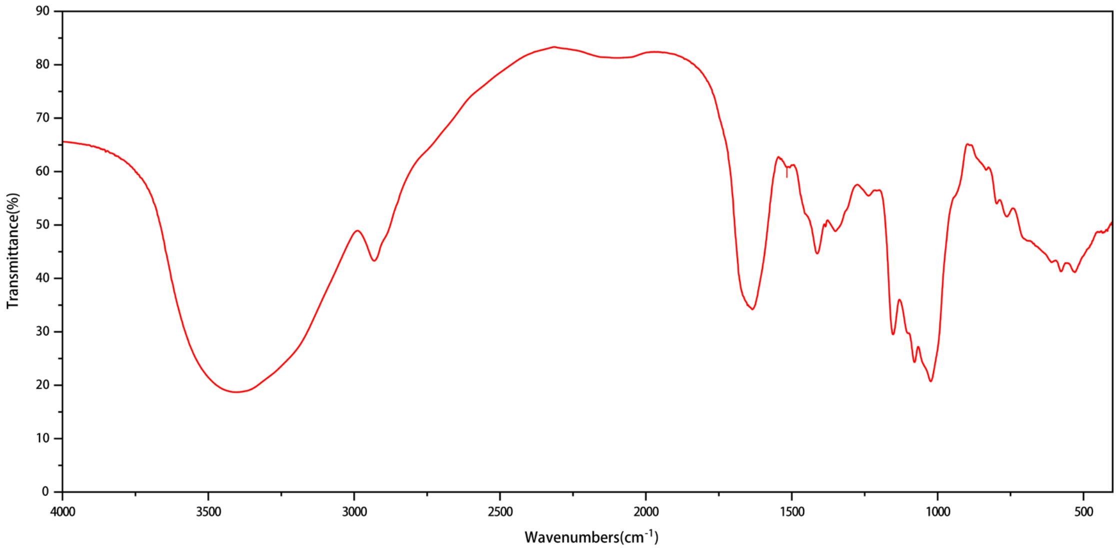


**Figure S2**. Fourier-transform infrared spectrum of PF40. The spectrum displayed characteristic absorption bands associated with polysaccharide functional groups, supporting the polysaccharide nature of PF40.

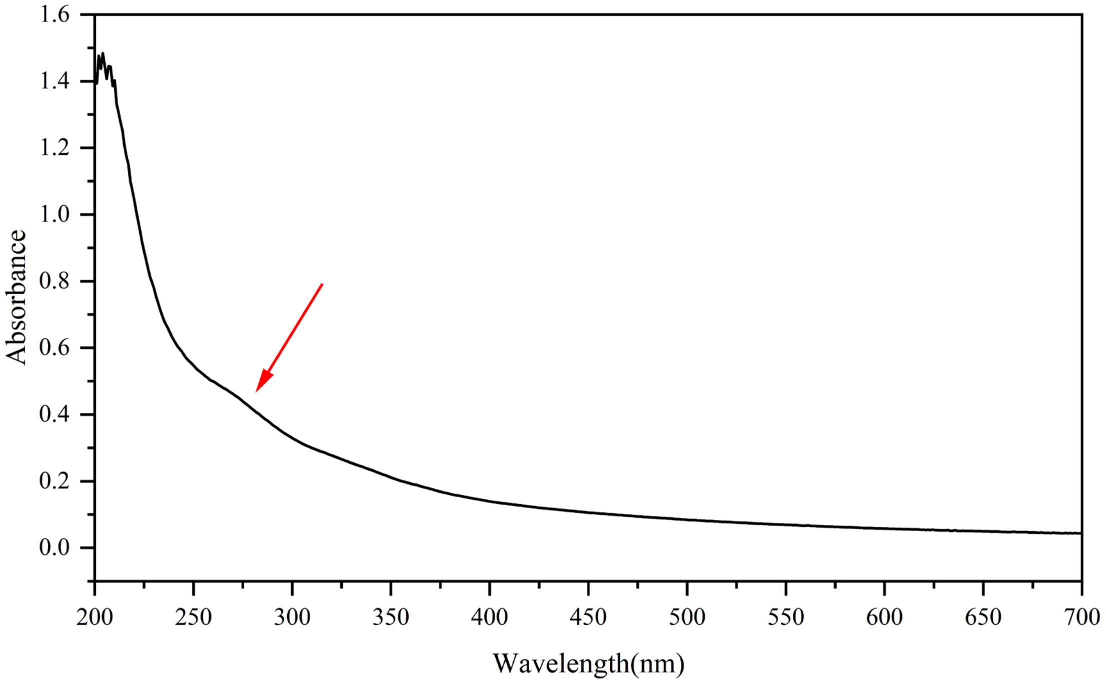


**Figure S3**. UV–visible absorption spectrum of PF40. PF40 showed no distinct absorption peaks at 260 or 280 nm, indicating minimal contamination by nucleic acids and proteins.

CON

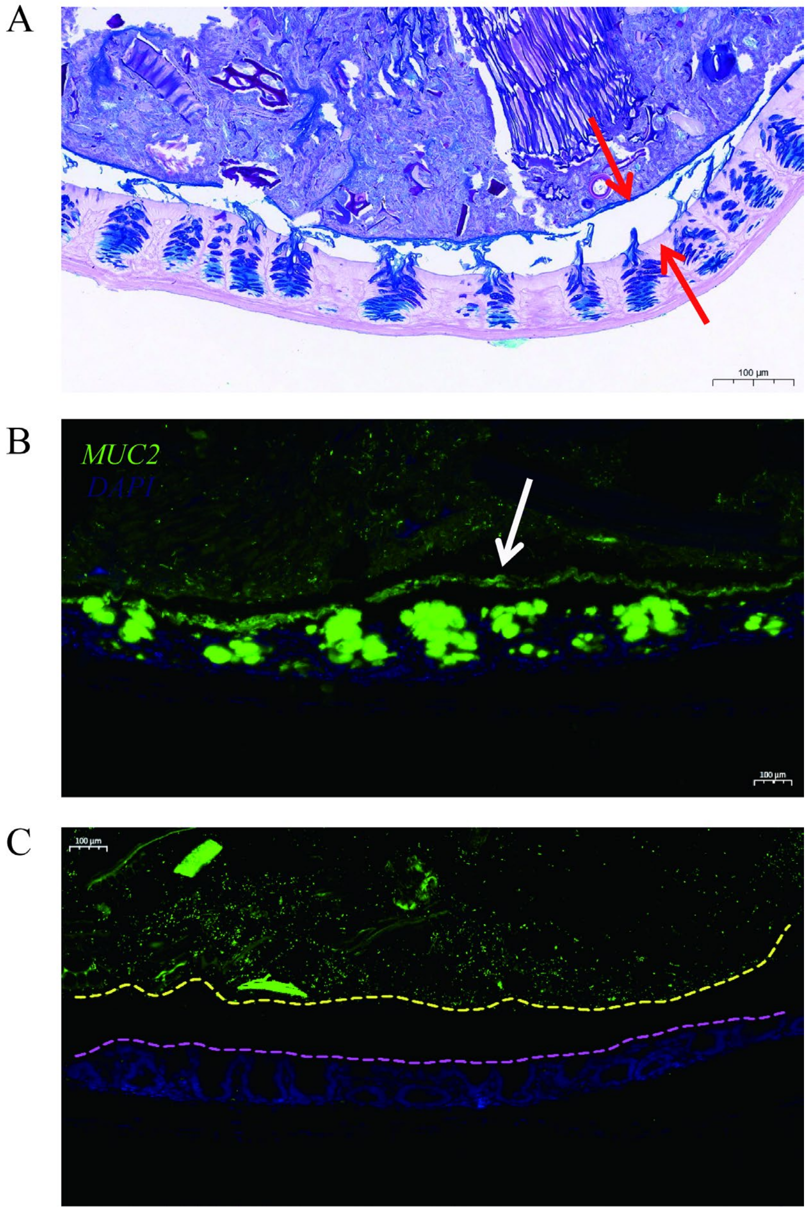


**Figure S4.** Separately presented CON reference images. (A) AB/PAS-stained luminal mucus band. (B) MUC2 immunofluorescence (green) and DAPI (blue). (C) EUB338-FISH signals (green) and DAPI (blue), with the two signal/epithelial boundaries indicated. Scale bars, 100 μm. These images provide descriptive context only; the reference feed was not compositionally matched to XTHF60.

CON

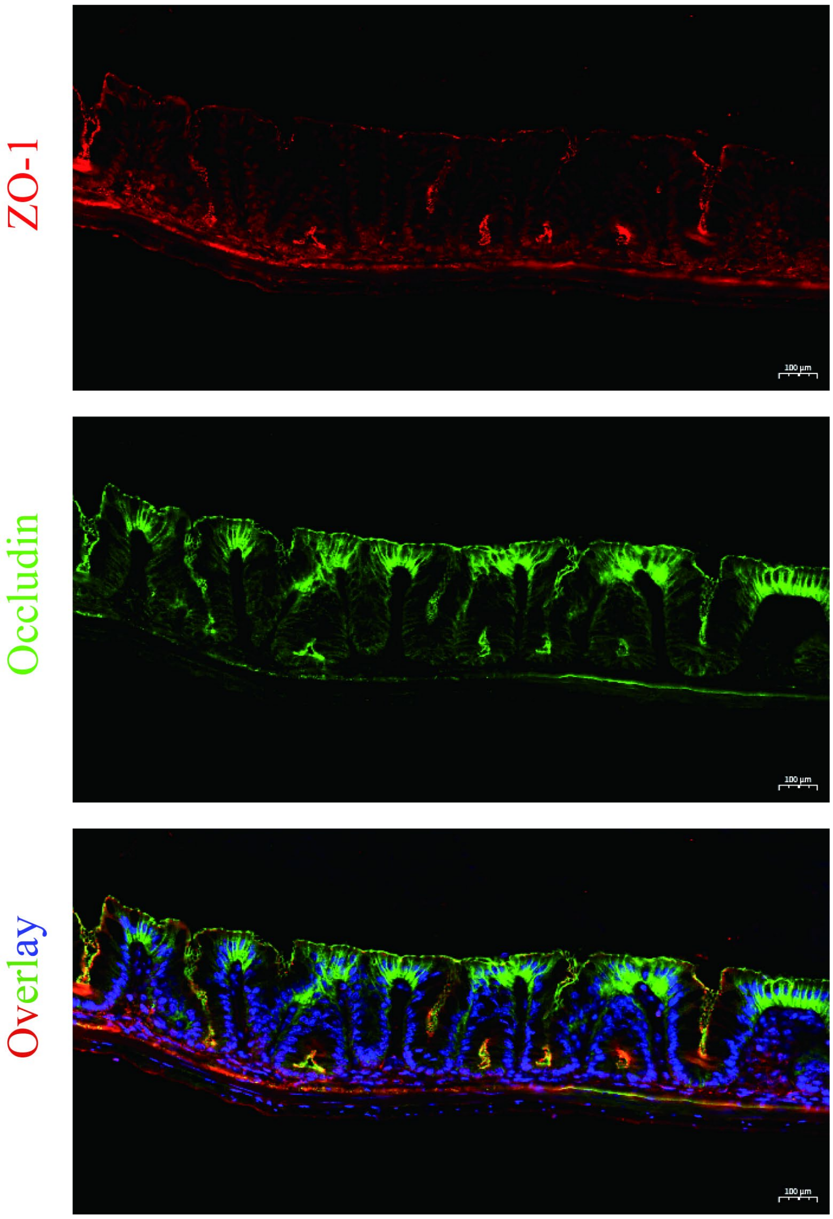


**Figure S5.** Separately presented CON reference immunofluorescence. ZO-1 is red, occludin is green and DAPI-stained nuclei are blue. Scale bars, 100 μm. These reference images are not used for inferential comparisons with MOD or PF40.

# Supplementary Tables

**Supplementary Table S1. Glucose metabolism parameters.**

| Parameter | CON reference | MOD | PF40 |
|---|---|---|---|
| Fasting glucose (mmol/L) | 5.53 ± 0.27 | 21.23 ± 3.42 | 15.43 ± 3.69 |
| Fasting insulin (μIU/mL) | 12.49 ± 0.72 | 22.95 ± 2.06 | 20.27 ± 1.85 |
| OGTT AUC (mmol·min/L) | 1346.70 ± 41.66 | 2432.10 ± 92.76 | 2308.80 ± 57.37 |
| HOMA-IR | 3.07 ± 0.21 | 21.68 ± 3.99 | 14.07 ± 4.23 |

Values are mean ± SD. Main-cohort n: CON-main, 7; MOD-main, 6; PF40-main, 7. Exception: OGTT AUC: n = 5 per group. CON is reported descriptively; no inferential CON contrasts are presented.

OGTT, oral glucose tolerance test; AUC, area under the curve; HOMA-IR, homeostatic model assessment of insulin resistance.

**Supplementary Table S2. Mucus barrier morphology and relative gene expression.**

| Parameter | CON reference | MOD | PF40 |
|---|---|---|---|
| Mucus band thickness (μm) | 35.54 ± 10.17 | 15.23 ± 5.79 | 31.60 ± 12.01 |
| AB/PAS-positive area (%) | 38.98 ± 5.78 | 29.91 ± 5.25 | 30.43 ± 2.74 |
| MUC2-positive area (%) | 24.14 ± 6.67 | 9.68 ± 3.68 | 20.59 ± 6.83 |
| *Tff3* expression | 0.901 ± 0.343 | 0.424 ± 0.161 | 0.785 ± 0.298 |
| *Clca1* expression | 0.927 ± 0.267 | 0.341 ± 0.130 | 0.519 ± 0.197 |
| *Fcgbp* expression | 1.066 ± 0.218 | 0.449 ± 0.171 | 0.588 ± 0.139 |
| *Agr2* expression | 0.956 ± 0.261 | 0.411 ± 0.131 | 0.768 ± 0.215 |
| Signal–epithelial distance (μm) | 118.36 ± 13.33 | 64.84 ± 13.03 | 85.49 ± 5.07 |

Values are mean ± SD. Main-cohort n: CON-main, 7; MOD-main, 6; PF40-main, 7. CON is reported descriptively; no inferential CON contrasts are presented.

AB/PAS, Alcian blue/periodic acid–Schiff. Gene expression values are relative mRNA levels ($2^{-\Delta\Delta Ct}$).

**Supplementary Table S3. Intestinal barrier and permeability parameters.**

| Parameter | CON reference | MOD | PF40 |
|---|---|---|---|
| ZO-1-positive area (%) | 21.90 ± 6.68 | 10.42 ± 1.97 | 13.75 ± 3.61 |
| Occludin-positive area (%) | 17.13 ± 3.83 | 3.86 ± 1.26 | 7.66 ± 3.22 |
| Serum FITC–dextran (μg/mL) | 0.563 ± 0.067 | 1.418 ± 0.094 | 0.967 ± 0.110 |
| Serum LPS (ng/mL) | 0.719 ± 0.074 | 2.392 ± 0.322 | 1.746 ± 0.247 |
| D-lactate (μmol/mL) | 0.117 ± 0.011 | 0.244 ± 0.029 | 0.209 ± 0.019 |
| DAO (U/L) | 184.23 ± 12.22 | 435.83 ± 59.23 | 352.44 ± 44.32 |

Values are mean ± SD. Main-cohort n: CON-main, 7; MOD-main, 6; PF40-main, 7. Exception: FITC–dextran: separate CON-FD/MOD-FD/PF40-FD cohorts, n = 3 per group. CON is reported descriptively; no inferential CON contrasts are presented.

FITC, fluorescein isothiocyanate; LPS, lipopolysaccharide; DAO, diamine oxidase.

**Supplementary Table S4. Inflammatory cytokine and chemokine levels.**

| Parameter | CON reference | MOD | PF40 |
|---|---|---|---|
| MCP-1 (pg/mg protein) | 975.93 ± 142.63 | 1997.00 ± 263.43 | 1566.24 ± 190.48 |
| TNF-α (pg/mg protein) | 69.68 ± 5.95 | 186.30 ± 37.38 | 133.66 ± 20.25 |
| IL-1β (pg/mg protein) | 282.76 ± 29.18 | 931.67 ± 90.65 | 668.29 ± 62.49 |
| IL-6 (pg/mg protein) | 104.53 ± 14.88 | 450.00 ± 102.13 | 315.31 ± 50.32 |

Values are mean ± SD. Main-cohort n: CON-main, 7; MOD-main, 6; PF40-main, 7. CON is reported descriptively; no inferential CON contrasts are presented.

MCP-1, monocyte chemoattractant protein-1; TNF-α, tumor necrosis factor-α; IL, interleukin.

**Table S5. Post-randomization pretreatment FBG and body weight.**

| Group | n | FBG (mmol/L) | Body weight (g) |
|---|---|---|---|
| MOD | 9 | 15.18 ± 1.76 | 27.80 ± 1.38 |
| PF40 | 10 | 15.32 ± 1.51 | 27.69 ± 1.66 |

Study week 6, before the first dose. Values are mean ± SD for all allocated animals, including the main and separate FITC–dextran cohorts.

**Table S6. Weekly body weight during treatment.**

| Treatment week | CON | MOD | PF40 |
|---|---|---|---|
| 0 | 26.13 ± 1.30 | 27.80 ± 1.38 | 27.69 ± 1.66 |
| 1 | 26.51 ± 1.34 | 28.05 ± 1.50 | 27.96 ± 1.63 |
| 2 | 26.85 ± 1.36 | 28.39 ± 1.56 | 28.28 ± 1.57 |
| 3 | 27.07 ± 1.38 | 28.74 ± 1.64 | 28.50 ± 1.37 |
| 4 | 27.36 ± 1.31 | 28.90 ± 1.50 | 28.87 ± 1.53 |
| 5 | 27.66 ± 1.42 | 29.10 ± 1.60 | 29.11 ± 1.54 |
| 6 | 28.05 ± 1.32 | 29.25 ± 1.60 | 29.33 ± 1.47 |
| Final gain | 1.92 ± 0.42 | 1.45 ± 0.49 | 1.64 ± 0.59 |

Values are mean ± SD in grams for all allocated animals (CON, n = 10; MOD, n = 9; PF40, n = 10). Treatment week 0 is the pretreatment baseline at study week 6; treatment weeks 1–6 correspond to study weeks 7–12. Final gain is the final weight minus pretreatment weight for each mouse. The exploratory MOD–PF40 Welch comparison gave $p = 0.4539$. CON is descriptive only. Food intake, energy intake and nutrient absorption were not measured or recorded.
